# Measurement of the operational efficiency of tertiary public hospitals in Western China: evidence from Guangxi from 2019 to 2022

**DOI:** 10.3389/fpubh.2025.1546402

**Published:** 2025-02-21

**Authors:** Junjie Huang, Haitao Yuan, Yanlong Wu, Huiyu Wang, Xinwen Liu, Bo Wei, Mengjian Zhang, Pinghua Zhu

**Affiliations:** ^1^School of Humanities and Social Sciences, Guangxi Medical University, Nanning, China; ^2^Liuzhou Workers Hospital, Liuzhou, China; ^3^Guangxi Zhuang Autonomous Region Medical Management Service Guidance Center, Nanning, China; ^4^School of Information and Management, Guangxi Medical University, Nanning, China; ^5^Liuzhou Municipal Liutie Central Hospital, Liuzhou, China; ^6^Guangxi Hospital Association, Nanning, China

**Keywords:** Guangxi tertiary public hospital, performance appraisal, operational efficiency, DEA-Malmquist, Tobit returns

## Abstract

**Objective:**

The continuous increase in health care costs and the growing demand for health services among residents make it an urgent priority to improve the operational efficiency of the health system in Chinese society. In this study, data on the operational efficiency of tertiary public hospitals in Guangxi were analyzed to identify issues in hospital management within the context of performance assessment, which thereby enhanced the social service capacity of hospitals.

**Methods:**

A comprehensive evaluation index system was constructed based on the “national monitoring indicators” of operational efficiency. The indicators were analyzed using data envelopment analysis with Banker, Charnes and Cooper (DEA-BCC) and Malmquist index models. The Tobit regression model was used to analyze the major factors affecting the efficiency of public hospitals.

**Results:**

Between 2019 and 2022, the pure technical efficiency (PTE) of 61 tertiary public hospitals in Guangxi remained at a relatively high level. The results of the Malmquist index showed a downward trend. The technical progress (TC) indicator became the main factor affecting the decline in the operational efficiency of hospitals. Tobit regression analysis revealed that plenty of factors exerted a significant impact on the operating efficiency of hospitals. These factors included the number of beds, the ratio of outpatient and inpatient patients relative to total patient numbers, the proportion of discharged patients undergoing surgery among total patients, business expenditures and total annual revenue.

**Conclusion:**

The scale of tertiary public hospitals in Guangxi is prominently unreasonable. It is necessary to raise the efficiency of resource utilization. The operation and management situation is not optimistic. Hospitals should accelerate the transformation of their development model, rationally allocate medical resources and shift from scale expansion to the improvement of quality and efficiency. Meanwhile, they should actively participate in establishing the hierarchical medical treatment system, controlling operating costs and reasonably increasing the proportion of personnel expenses to improve operational and management efficiency.

## 1 Introduction

Some countries including China are facing the dual challenges of rising health care costs and low hospital efficiency. Specifically, China is currently dealing with an accelerating aging population, a slowing population growth rate and a shift in the disease spectrum, all of which are driving an increasing demand for health services of high quality. Public hospitals, the primary providers of social health services, bear tremendous pressure in response to the growing demand for these services. Research by Hu et al. (1) reported that the health resource allocation and service provision in China are inefficient. The inequitable distribution of medical resources has potentially threatened social stability. Therefore, policymakers need to improve the utilization efficiency of health resources to enhance the social service capabilities of hospitals. Strengthening hospital operations management is crucial to ensure efficient medical resource utilization, achieve refined hospital management and promote a transition from scale expansion to quality and efficiency-oriented development. In 2019, a unified performance assessment indicator system for tertiary public hospitals was set up for the first time, which marked the beginning of a nationwide performance evaluation of these hospitals. The performance assessment of tertiary public hospitals serves as a “guiding rod” for the development of public hospitals. It is of great significance to expedite the establishment of a tiered diagnosis and treatment system, build a modern hospital management system and enhance the operational management capabilities of hospitals. In 2020, the “Guiding Opinions on Strengthening the Operational Management of Public Hospitals” was issued by the National Health Commission of China and the National Administration of Traditional Chinese Medicine. This document highlighted that strengthening the operational management of public hospitals is an important means of leading high-quality hospital development with new development concepts, deepening comprehensive reforms in public hospitals and alleviating economic operational pressure in these hospitals. This policy further elevated the operational management of public hospitals to a strategic development level. Health resources are significantly fewer in the western region of China compared with other regions (2). In Western China, hospitals have weaker operational management capabilities. It is of great importance to promote national unity with many methods. Such methods involve using limited health resource inputs to meet the growing demand for health services while keeping pace with deepening medical reforms, updating internal hospital management systems, addressing internal operational management weaknesses (3) and fairly allocating health resources. This is also a key issue of common research interest in both the Chinese academia and political arena.

At present, data envelopment analysis (DEA) is the most extensively used method for studying and evaluating the operational efficiency of hospitals (4). It was proposed by Charnes, a renowned American operations researcher, and other scholars in 1978, and later introduced by Nunamaker to the field of hospital management (5). This method can consider a variety of input and output indicators simultaneously and avoid evaluation difficulties caused by different units of measurement. Internationally, scholars have examined hospital efficiency from various dimensions. In terms of research at the national level, Afonso and Aubyn (6) used DEA to assess the health efficiency of 30 Organization for Economic Cooperation and Development (OECD) countries and considered efficiency scores with environmental variables. Top et al. (7) evaluated the efficiency of health systems in 36 African countries. Regarding research within the same country but across different regions, Mazon (8) assessed the technical efficiency of public health expenditures in the municipalities of Santa Catarina and their connection with health management outcomes. Ngobeni (9) measured and compared the technical efficiency of healthcare delivery across the nine provinces of South Africa. Bates (10) used DEA and multiple regression analysis to empirically examine the impact of multiple market structure factors on the technical efficiency of hospital services in major United States (US) metropolitan areas. Ferreira et al. (11–13) applied extended DEA models like log-DEA and bootstrap to analyze the Portuguese health care system. From the perspective of health resource control: Zhang et al. (14) analyzed tertiary public hospitals in nine provinces along the Yellow River and found that the Coronavirus Disease 2019 (COVID-19) pandemic greatly affected technological changes in hospitals. They proposed that government departments reasonably control the flow of health resources and that hospitals enhance the application of technology. Li et al. (15) statically analyzed 30 hospitals in Shanxi and concluded considerable redundancy in hospital inputs, which suggested a need to transform hospital management models to improve operational efficiency. From the angle of scale efficiency (SE): He et al. (16) analyzed tertiary public hospitals in Heilongjiang and concluded that “SE” is the primary factor causing the imbalance of hospital development. There is a need to further improve operational efficiency and avoid the unregulated expansion of hospitals. Qin et al. (17) analyzed the cross-sectional data of public hospitals in Hunan by use of the DEA-Tobit model and emphasized the necessity of improving the efficiency of public hospitals and strictly controlling the scale of tertiary hospitals.

This study is the first to combine the “national monitoring indicators” from the “Performance Assessment of Tertiary Public Hospitals” to investigate and evaluate the operational efficiency of hospitals in the western region of China. To be specific, it makes the following contributions: Firstly, existing studies have all focused on the overall level of a region and provided limited guidance for individual hospitals despite having used the DEA or Malmquist model to evaluate public hospitals. This study breaks away from the integrative evaluation framework of other papers and evaluates Guangxi tertiary public hospitals from a micro perspective. It presents the evaluation results of 61 hospitals, identifies hospitals with significant issues, and offers valuable insights into the management practices of other hospitals. Secondly, qualitative (18) and quantitative (19, 20) approaches were combined to select indicators. This scientific method avoids the pitfalls of relying on a single approach. Thirdly, based on DEA, the Tobit model was employed to perform regression analysis on the factors influencing comprehensive efficiency (CE), pure technical efficiency (PET) and SE. This provides a more comprehensive analysis and control of real-world issues affecting the operational efficiency of public hospitals. The present study provides evidence-based support for gradually improving the operational management model of tertiary public hospitals in the western border regions of China and promoting their high-quality social health services.

## 2 Materials and methods

### 2.1 Data source

The data were obtained from the statistical yearbooks and financial statements of 61 third-tier public hospitals located in the Guangxi Zhuang Autonomous Region and from the “Performance Evaluation of Third-Tier Public Hospitals” covering the years 2019–2022. The sample comprised 44 general hospitals and 17 specialized hospitals. Among them, 20 provincial hospitals, 36 municipal hospitals and five county-level hospitals were included. The sampled hospitals were systematically coded from H1 to H61.

### 2.2 Indicator system construction

**Figure 1:**
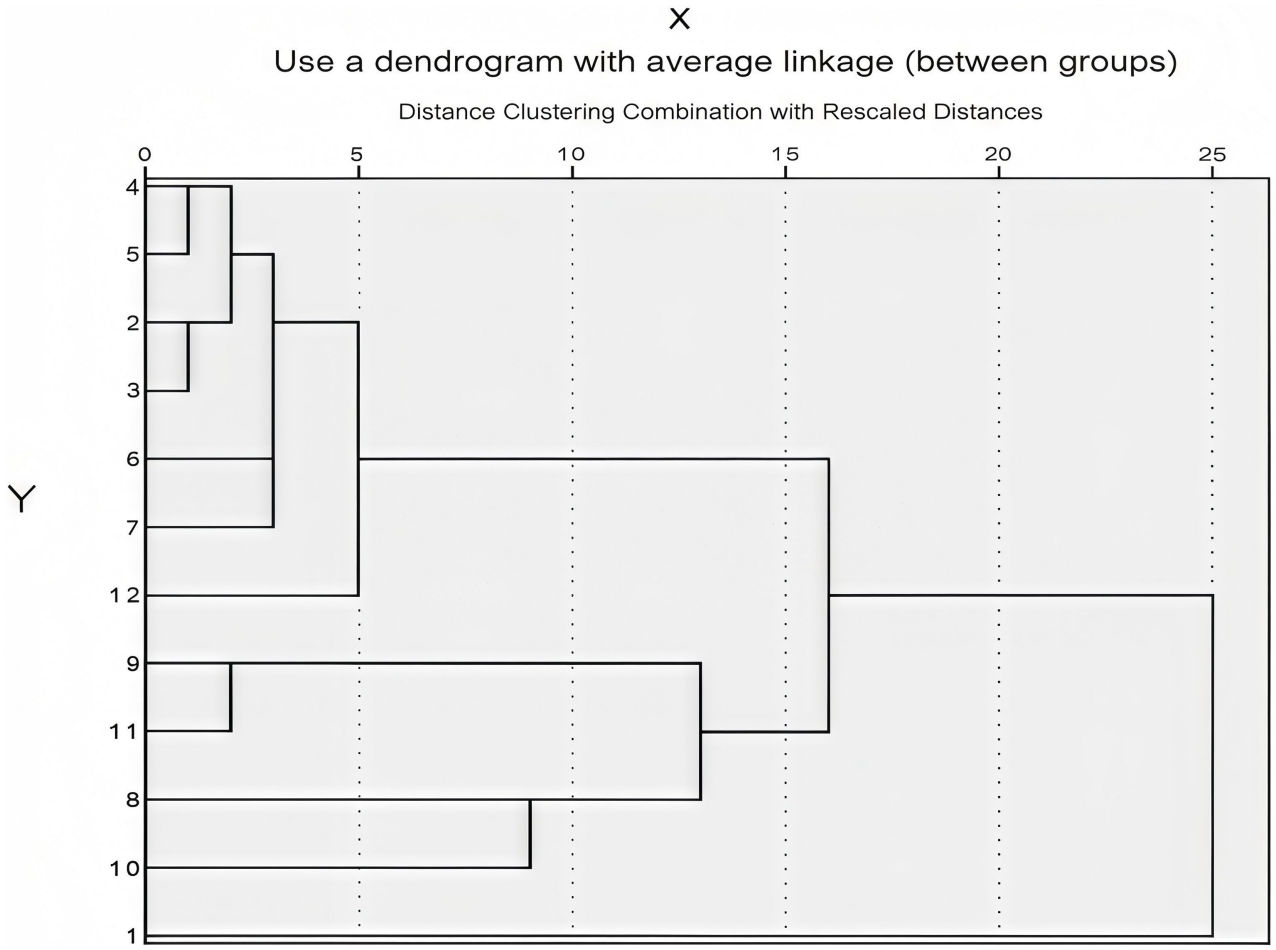
Clustering results of input indicator library. The Y-axis from 1 to 12 corresponds to the indicators X1 to X12 in the indicator library, respectively.

According to an important empirical rule of DEA theory, the number of indicators in DEA studies should be considered concerning the number of decision-making units (DMUs). The inclusion of too many indicators in the model can lead to insensitive efficiency results, low differentiation and ineffective evaluations. Thus, the number of DMUs is supposed to be at least two to three times the sum of input and output indicators (19, 20). Based on the three-dimensional approach of human resource, physical resource and cost inputs developed by Ozcan et al. (21), an input indicator library related to operational efficiency from the “National Tertiary Public Hospital Performance Assessment Manual (2024 Edition)” was constructed in this study, as detailed in Table 1 (22–27). Cluster analysis was used to evaluate the indicators in the input indicator library (Figure 1), to minimize the collinearity effects among indicators of the same type (18, 28). Ultimately, it was concluded that the indicator representing the human resource input dimension is X3: the ratio of doctors to nurses; the physical resource input dimension is denoted by X4: the number of hospital beds; the cost input dimension is indicated by X8: average outpatient medical expenses per visit; X9 represents average outpatient drug expenses per patient. Output indicators were selected based on the “National Tertiary Public Hospital Performance Assessment Manual (2024 Edition) (29).” Revenue and expenditure structure and cost control were used as second-level indicators, and nine national monitoring indicators were taken as third-level indicators (Table 2). The DEAP 2.1 software was utilized for analyzing the input–output indicators of sample hospitals, and the operational efficiency of tertiary public hospitals in Guangxi was evaluated systematically. Additionally, indicators that may affect the static efficiency of hospitals were chosen based on the four dimensions of operational efficiency, sustainable development, medical quality and satisfaction from the “National Assessment” (Table 3). Tobit regression analysis was performed using the Stata 18 software (30–34).

**Table 1:** Hospital resource input indicator library.

Category of indicator	Evaluation indicators
Human resource input	X1:number of physicians;X2:number of nurses;X3:ratio of doctors to nurses
Physical resource input	X4:number of hospital beds;X5:annual energy consumption of hospitals
Cost input	X6:medical staff expenditure; X7:medical expenditure; X8:outpatient average medical expenses per visit; X9:average outpatient drug expenses per patient; X10:average medical expenses per discharged patient; X11:average outpatient drug expenses per patient; X12:assets of hospitals

**Table 2:** Input-output indicator system for operational efficiency of tertiary public hospitals in Guangxi.

Primary Indicators	Secondary indicators	Third-level indicators	Explanation of indicators	Indicator attributes
Input indicators	Human resource input	Ratio of doctors to nurses	The ratio of medical personnel to patients in a hospital or medical institution.	Positive indicators
	Physical resource input	Number of hospital beds	The actual number of beds open in a hospital refers to the fixed number of beds that are actually available at the end of the year.	Positive indicators
	cost input	Outpatient average medical expenses per visit	The average cost of medical services for outpatient patients per visit	Negative indicators
		verage medical expenses per discharged patient	The average cost of medical expenses for discharged patients per hospitalization	Negative indicators
Output indicators	Income and expenditure structure	The proportion of revenue from medical services	Medical service revenue÷medical revenue×100%	Positive indicators
		Percentage of personnel expenses	Personnel expenses÷Medical activity expenses×100%	Positive indicators
		Annual energy consumption share	Annual total energy consumption÷annual total revenue×10,000	Negative indicators
		Medical surplus ratio	Medical surplus÷Medical activity revenue×100%	Interval Indicator
		Debt-to-Asset Ratio	Total Liabilities÷Total Assets×100%	Interval Indicator
	Cost Control	The average increase in outpatient consultation fees	(The average medical expenses for outpatient patients this year—the average medical expenses for outpatient patients last year)÷the average medical expenses for outpatient patients last year×100%	Negative indicators
		Increase in the average cost of prescription drugs per outpatient visit	(Average Prescription Cost per Outpatient Visit this Year—Average Prescription Cost per Outpatient Visit Last Year)÷Average Prescription Cost per Outpatient Visit Last Year×100%	Negative indicators
		Increase in the average cost per hospitalization	(Average Cost of Medical Services per Discharged Patient in the Current Year—Average Cost of Medical Services per Discharged Patient in the Previous Year)÷Average Cost of Medical Services per Discharged Patient in the Previous Year×100%	Negative indicators
		Average Inpatient Drug Cost Increase	(Average Per Patient Drug Cost for Discharged Patients in the Current Year—Average Per Patient Drug Cost for Discharged Patients in the Previous Year)÷Average Per Patient Drug Cost for Discharged Patients in the Previous Year×100%	Negative indicators

**Table 3:** Estimation results of static efficiency of 61 third-tier public hospitals in Guangxi province using Tobit model.

Dimension of Indicator	Explanatory variable	CE (95% confidence interval)	PTE (95% confidence interval)	SE (95% confidence interval)
Functional positioning	Bed size scale	0.0428** (0.0151,0.0705)	0.1036** (0.0151,0.1920)	0.0275** (0.0066,0.0485)
	Jurisdictional Level	−0.0161 (−0.0408,0.0086)	0.0175 (−0.0373,0.0724)	−0.0177* (0.0368,0.0013)
	Ratio of outpatient to inpatient patients	0.0014* (−0.0001,0.0029)	0.0019 (−0.0037,0.0075)	0.0010* (−0.0000,0.0021)
	The proportion indicator of discharged patients undergoing surgery	−0.0033** (−0.0059,-0.0008)	−0.0013 (−0.0070,0.0043)	−0.0027** (−0.0047,-0.0006)
Operational efficiency	Business expenses	−0.0000** (−0.0000,0.0001)	−0.0000 (−0.0000,0.0001)	−0.0000** (−0.0000,0.0001)
	annual total revenue	0.0000** (−0.0000,0.0001)	0.0000 (−0.0000,0.0001)	0.0000** (−0.0000,0.0001)
Sustainable development	The structure of healthcare professionals’ ranks	0.0182 (−0.1269,0.1636)	0.1436 (−0.2102,0.4974)	−0.0042 (−0.1152,0.1067)
Satisfaction	Satisfaction of healthcare workers	0.0015 (−0.0010,0.0040)	0.0029 (−0.0030,0.0089)	0.0008 (−0.0010,0.0028)
	Patient Satisfaction in Outpatient Department	−0.0008 (−0.0072,0.0056)	−0.0024 (0.0179,0.0130)	0.0000 (−0.0048,0.0049)
	Satisfaction of Inpatient Patients	−0.0039 (−0.0129,0.0051)	0.0087 (−0.0124,0.0299)	−0.0041 (0.0110,0.0027)
	Constant	1.3197 (0.6151,2.0242)	0.0045 (−1.608,1.6178)	1.3540 (0.8079,1.9001)

* indicates *p* < 0.1, ** indicates *p* < 0.

In the context of DEA analysis, it is essential to conduct positive and standardized processing when interval and negative indicators within the input–output indicator system are addressed. The majority of articles in this field employ the reciprocal method to transform negative indicators into positive ones. This non-linear approach has the potential to alter the distribution characteristics of original data (35). Hence, the Min-Max reverse indicator formula (Equation 1) was used in this study to process negative indicators (36), where X_ij_ stands for the original value of the j indicator for the i evaluation object. The medical surplus rate and asset-liability ratio were processed using the interval-type attribute standardization method with the standardization formula (Equation 2) (16). In the formula, [a,b] represents the optimal attribute interval; $a_{j}^{0}$ stands for the intolerable lower limit; $a_{j}^{*}$ denotes the intolerable upper limit, with $b_{ij}$ converted maximum value being 1 and the minimum value being 0.


(1) $$x_{ij}=\frac{\max_{i}X_{ij}-X_{ij}}{\max_{i}X_{ij}-\min_{i}X_{ij}}$$

(2) $$b_{ij}=\{\begin{array}{c} 1-\frac{\left(c-a_{ij}\right)}{\left(c-a_{j}^{0}\right)},a_{ij}\in [a_{j}^{0},c)\\ 1,a_{ij}\in \left[c,d\right]\;\\ \;1-\frac{\left(a_{ij}-d\right)}{\left(a_{j}^{*}-d\right)},a_{ij}\in (d,a_{j}^{*}]\\ 0,\mathrm{else}\; \end{array}$$

### 2.3 Methods

#### 2.3.1 DEA-BCC model

Common DEA static models include the Charnes, Cooper and Rhodes (CCR) model measuring constant returns to scale (CRS) and the Banker, Charnes and Cooper (BCC) model assessing variable returns to scale (VRS). Health production theory suggests that the production technology within health systems is characterized by VRS. Consequently, the BCC model was used in the current study for static analysis of sample hospitals (37). The BCC model enables the calculation of CE and returns to scale (RTS) in hospital operations. CE can be further decomposed into PTE and SE, which is represented by the equation CE = PTE × SE. The BCC model incorporates convexity constraints and reflects its assumption of VRS, which is opposite to the CCR model. Below is the linear programming formulation (Equation 3):


(3) $$\left(BCC\right)\{\begin{array}{c} \min\theta =V_{D},\\ s.t.\overset{n}{\underset{j=1}{\sum }}x_{j}\lambda_{j}+s^{-}=\theta x_{0},\\ \overset{n}{\underset{j=1}{\sum }}y_{j}\lambda_{j}-s^{-}=y_{0},\\ \overset{n}{\underset{j=1}{\sum }}\lambda_{j}=1,\\ s^{-}\geq 0,s^{+}\geq 0,\lambda_{j}\geq 0,j=1,2,\cdots ,n\text{.} \end{array}$$

The optimal solution of the linear programming problem is described as follows:

If θ ^0^ = 1 and $s^{-}$ =0, $s^{+}$ =0, the DMU DMU_j0_ is DEA efficient. In this case, its production activities have SE and technical efficiency.

If θ ^0^ = 1 and s $=s^{-}$ + $s^{+}$ >0, DMU_j0_ is slightly inefficient according to DEA. In this case, its production activities are not simultaneously efficient in terms of SE and technical efficiency.

When θ ^0^ < 1, DMU_j0_ is considered DEA inefficient. In this case, its production activities have no SE and technical efficiency.

#### 2.3.2 Malmquist index model

In 1953, the Malmquist index was initially introduced by Sten Malmquist, a Swedish economist, to analyze variations in consumption indices across different periods (38). Subsequently, this concept was adapted by Caves for production analysis, to measure dynamic efficiency changes in production activities. On this basis, Fare and colleagues developed the Malmquist index by employing the geometric average of the indices from two consecutive periods to assess the trend of total factor productivity (TFP) changes from a specific period t to t + 1 (39). The model is represented as follows (Equation 4).


(4) $$M\left(x^{t},y^{t},x^{t+1},y^{t+1}\right)={\left[\frac{D^{t}\left(x^{t+1},y^{t+1}\right)}{D^{t}\left(x^{t},y^{t}\right)}\times \frac{D^{t+1}\left(x^{t+1},y^{t+1}\right)}{D^{t+1}\left(x^{t},y^{t}\right)}\right]}^{\frac{1}{2}}$$

TFP is influenced simultaneously by two key factors: technical efficiency change (TEC) and technology progress (TC), which can be expressed as TFP = TC × TEC. Furthermore, TEC can be further broken down into the product of PTE change (PTEC) and SE change (SEC). Thus, the expression for TFP can also be articulated as TFP = TC × PTEC × SEC.

The Malmquist index has a threshold value of 1:

M = 1 indicates that efficiency remains unchanged.

M > 1 indicates that the efficiency in period t + 1 is on an upward trend compared to that in period t.

M < 1 indicates that the efficiency in period t + 1 is on a downward trend compared to that in period t.

#### 2.3.3 Tobit model

In the process of performing regression analysis, continuous dependent variables may sometimes be constrained to a specific range due to truncation or censoring, which can result in inconsistent estimators. As defined by Davidson et al., truncation means the systematic exclusion of certain observations from the sample, whereas censoring refers to a scenario where no observations are excluded but some are limited to a particular threshold. Both truncated and censored variables are collectively termed restricted dependent variables (40, 41). Also called the censored or truncated regression model, the Tobit model is frequently employed in regression analyses involving restricted variables. Its fundamental form is represented as follows (Equation 5):


(5) $$y_{i}=\alpha +\beta x_{i}+\upsilon_{i}$$

In this study, dependent variables including CE, PTE and SE exhibited a value range of (0, 1), and were categorized as censored data. Consequently, the Tobit model was employed for analyzing the influence factors for the static efficiency of sample hospitals and exploring both the direction and magnitude of these effects. The model can be formulated as follows (Equation 6):


(6) $$\begin{array}{l} \mathrm{EFFit}=\alpha +X1\mathrm{it}+X2\mathrm{it}+X3\mathrm{it}+X4\mathrm{it}+X5\mathrm{it}+\\ X6\mathrm{it}+X7\mathrm{it}+X8\mathrm{it}+X9\mathrm{it}+X10\mathrm{it}+\\ X11\mathrm{it}+X12\mathrm{it}+X13\mathrm{it}+\ldots \ldots + \end{array}$$

Where EFFit represents the operational efficiency of the pilot hospital; α stands for the constant term; *β* denotes the coefficient for the impact of independent variables on efficiency; i refers to the serial number of each observation (1, 2, 3, …, 61); t indicates the year (2019, 2020, 2021 and 2022); *ϵ* is the random disturbance term.

## 3 Results

### 3.1 Static analysis of the BCC model in third-tier public hospitals in Guangxi

As shown in Table 4, CE serves as an indicator of the production capacity of a hospital within the healthcare industry (42). From 2019 to 2022, the proportion of DEA-effective hospitals (those with a CE value of 1) among the 61 tertiary public hospitals in Guangxi was recorded at 49.18, 31.15, 36.07 and 39.34%, respectively, which demonstrated an initial decline followed by a gradual recovery trend. Only 12 hospitals (19.67%) maintained DEA-effectiveness throughout the four years, including six in Nanning, two in Liuzhou and one each in Wuzhou, Yulin, Baise and Laibin. Notably, over 80% of these hospitals did not operate on the efficient frontier, which indicated the failure of their resource inputs to achieve optimal output levels relative to best practices. PTE, which assumes CRS, quantifies the gap between the production capacity and the frontier of a hospital. This metric is an indicator of both technological proficiency and managerial competence. Over four years, 14 hospitals (22.95%) achieved a DEA value of 1 for PTE. Moreover, 27 hospitals experienced fluctuations in performance but successfully returned to the production frontier in 2022. The overall strong performance of the sampled hospitals was closely tied to the robust initiatives of the region that were aimed at advancing healthcare system reforms. Given the current level of technology, SE assesses the disparity between the actual and optimal production scale of a hospital. This metric is instrumental in determining whether a hospital should expand or contract its operations to enhance operational efficiency (43). During the investigation period, 36 hospitals (59.01%) exhibited a fluctuating decline in SE. The proportion of hospitals demonstrating DEA-inefficient SE was recorded at 50.82, 68.85, 63.93 and 60.66% over the four years. Of note, only 12 hospitals (19.67%) achieved a DEA value of 1 for SE throughout this period, which underscored the issue of irrational production scale within hospitals. Decreasing Returns to Scale (DRS) refers to the variation in output that occurs when the proportions of various production factors within a hospital are altered, while other conditions remain constant. In the past four years, the number of hospitals experiencing DRS was recorded as 31 (50.82%), 36 (59.02%), 39 (63.93%) and 35 (57.38%), respectively, which indicated a need for adjustments in their production scale.

**Figure 2:**
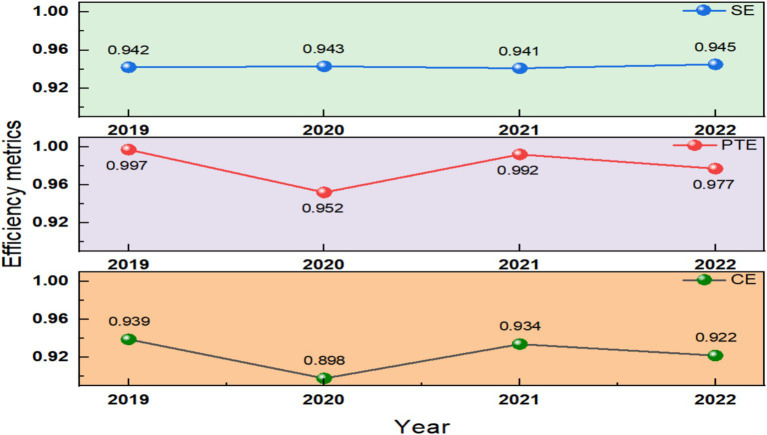
Comparison of average static decomposition indicators from 2019 to 2022.

The trend of mean values for SE, PTE and CE from 2019 to 2022 is illustrated in Figure 2.

**Table 4:** Static decomposition of 61 tertiary public hospitals in Guangxi from 2019 to 2022 unit: [*n*(%)].

Hospital	2019				2020				2021				2022			
	TCE	PTE	SE	RTS	TCE	PTE	SE	RTS	TCE	PTE	SE	RTS	TCE	PTE	SE	RTS
H1	1.000	1.000	1.000	-	1.000	1.000	1.000	-	1.000	1.000	1.000	-	1.000	1.000	1.000	-
H2	0.919	1.000	0.919	drs	0.810	0.858	0.944	drs	1.000	1.000	1.000	-	0.994	1.000	0.994	drs
H3	1.000	1.000	1.000	-	1.000	1.000	1.000	-	1.000	1.000	1.000	-	1.000	1.000	1.000	-
H4	0.876	1.000	0.876	drs	0.911	1.000	0.911	drs	0.917	1.000	0.917	drs	0.750	1.000	0.750	drs
H5	1.000	1.000	1.000	-	0.914	1.000	0.914	irs	1.000	1.000	1.000	-	1.000	1.000	1.000	-
H6	1.000	1.000	1.000	-	1.000	1.000	1.000	-	1.000	1.000	1.000	-	1.000	1.000	1.000	-
H7	1.000	1.000	1.000	-	1.000	1.000	1.000	-	1.000	1.000	1.000	-	1.000	1.000	1.000	-
H8	1.000	1.000	1.000	-	1.000	1.000	1.000	-	1.000	1.000	1.000	-	1.000	1.000	1.000	-
H9	1.000	1.000	1.000	-	0.976	0.989	0.987	irs	0.986	1.000	0.986	drs	0.833	0.866	0.962	drs
H10	1.000	1.000	1.000	-	0.813	0.896	0.907	drs	0.868	1.000	0.868	drs	0.814	1.000	0.814	drs
H11	0.846	1.000	0.846	drs	0.848	0.919	0.923	drs	0.906	1.000	0.906	drs	1.000	1.000	1.000	-
H12	1.000	1.000	1.000	-	0.941	0.942	0.998	irs	1.000	1.000	1.000	-	1.000	1.000	1.000	-
H13	0.931	1.000	0.931	drs	0.860	1.000	0.860	drs	0.980	1.000	0.980	drs	1.000	1.000	1.000	-
H14	0.869	1.000	0.869	drs	0.768	0.821	0.935	drs	0.920	1.000	0.920	drs	0.917	1.000	0.917	drs
H15	1.000	1.000	1.000	-	1.000	1.000	1.000	-	1.000	1.000	1.000	-	1.000	1.000	1.000	-
H16	1.000	1.000	1.000	-	1.000	1.000	1.000	-	0.858	1.000	0.858	drs	0.841	1.000	0.841	drs
H17	0.776	1.000	0.776	drs	0.636	0.791	0.804	drs	0.758	0.908	0.835	drs	0.840	1.000	0.840	drs
H18	0.956	1.000	0.956	drs	0.816	0.959	0.851	drs	0.680	1.000	0.680	drs	0.728	1.000	0.728	drs
H19	0.856	1.000	0.856	drs	0.796	0.889	0.895	drs	0.819	1.000	0.819	drs	0.855	1.000	0.855	drs
H20	1.000	1.000	1.000	-	0.797	0.870	0.916	drs	0.846	1.000	0.846	drs	0.892	1.000	0.892	drs
H21	1.000	1.000	1.000	-	1.000	1.000	1.000	-	1.000	1.000	1.000	-	1.000	1.000	1.000	-
H22	1.000	1.000	1.000	-	1.000	1.000	1.000	-	1.000	1.000	1.000	-	1.000	1.000	1.000	-
H23	0.841	1.000	0.841	drs	0.876	0.958	0.915	drs	0.953	1.000	0.953	drs	0.888	1.000	0.888	drs
H24	0.800	0.993	0.806	drs	0.813	0.935	0.870	drs	0.789	1.000	0.789	drs	0.806	0.893	0.903	drs
H25	0.796	1.000	0.796	drs	0.758	0.900	0.842	drs	0.840	0.947	0.887	drs	0.823	1.000	0.823	drs
H26	0.890	1.000	0.890	drs	1.000	1.000	1.000	-	0.965	1.000	0.965	drs	1.000	1.000	1.000	-
H27	1.000	1.000	1.000	-	0.892	0.908	0.982	drs	0.976	1.000	0.976	drs	1.000	1.000	1.000	-
H28	0.886	1.000	0.886	drs	0.921	0.974	0.945	drs	0.819	1.000	0.819	drs	1.000	1.000	1.000	-
H29	0.873	0.998	0.876	drs	1.000	1.000	1.000	-	0.949	1.000	0.949	drs	0.822	0.832	0.988	drs
H30	0.851	1.000	0.851	drs	0.834	0.884	0.943	drs	0.839	1.000	0.839	drs	0.934	1.000	0.934	drs
H31	0.933	1.000	0.933	drs	0.975	0.995	0.980	drs	0.955	0.973	0.981	drs	1.000	1.000	1.000	-
H32	1.000	1.000	1.000	-	1.000	1.000	1.000	-	1.000	1.000	1.000	-	1.000	1.000	1.000	-
H33	1.000	1.000	1.000	-	1.000	1.000	1.000	-	1.000	1.000	1.000	-	0.999	1.000	0.999	irs
H34	1.000	1.000	1.000	-	0.984	0.985	0.999	-	0.935	1.000	0.935	drs	0.897	1.000	0.897	drs
H35	0.991	1.000	0.991	drs	0.888	0.918	0.967	drs	0.938	0.950	0.988	drs	0.874	0.890	0.982	drs
H36	0.903	1.000	0.903	drs	0.806	0.836	0.964	drs	0.998	1.000	0.998	drs	0.650	0.658	0.988	drs
H37	0.856	1.000	0.856	drs	0.859	1.000	0.859	drs	0.895	1.000	0.895	drs	0.940	1.000	0.940	drs
H38	1.000	1.000	1.000	-	0.756	1.000	0.756	drs	1.000	1.000	1.000	-	0.788	1.000	0.788	drs
H39	0.939	1.000	0.939	drs	0.741	0.781	0.949	drs	0.938	1.000	0.938	drs	0.899	1.000	0.899	drs
H40	1.000	1.000	1.000	-	0.881	1.000	0.881	drs	0.908	1.000	0.908	drs	0.997	1.000	0.997	drs
H41	0.949	1.000	0.949	drs	0.867	0.913	0.950	drs	0.963	1.000	0.963	drs	0.827	0.840	0.985	drs
H42	0.760	0.847	0.897	drs	1.000	1.000	1.000	-	0.941	1.000	0.941	drs	0.886	1.000	0.886	drs
H43	1.000	1.000	1.000	-	1.000	1.000	1.000	-	1.000	1.000	1.000	-	1.000	1.000	1.000	-
H44	1.000	1.000	1.000	-	0.952	1.000	0.952	drs	1.000	1.000	1.000	-	1.000	1.000	1.000	-
H45	1.000	1.000	1.000	-	0.941	0.948	0.992	irs	1.000	1.000	1.000	-	0.990	1.000	0.990	drs
H46	1.000	1.000	1.000	-	1.000	1.000	1.000	-	1.000	1.000	1.000	-	1.000	1.000	1.000	-
H47	1.000	1.000	1.000	-	0.851	1.000	0.851	drs	0.883	1.000	0.883	drs	0.856	1.000	0.856	drs
H48	0.801	1.000	0.801	drs	0.706	0.870	0.812	drs	0.743	0.885	0.840	drs	1.000	1.000	1.000	-
H49	0.897	1.000	0.897	drs	0.844	0.869	0.971	drs	0.887	1.000	0.887	drs	0.909	1.000	0.909	drs
H50	1.000	1.000	1.000	-	1.000	1.000	1.000	-	0.986	1.000	0.986	drs	1.000	1.000	1.000	-
H51	0.976	1.000	0.976	drs	1.000	1.000	1.000	-	0.885	0.903	0.980	drs	0.826	0.830	0.995	irs
H52	1.000	1.000	1.000	-	1.000	1.000	1.000	-	1.000	1.000	1.000	-	1.000	1.000	1.000	-
H53	0.896	1.000	0.896	drs	0.816	0.817	0.999	drs	0.959	0.987	0.972	drs	0.978	1.000	0.978	drs
H54	0.832	1.000	0.832	drs	0.852	1.000	0.852	drs	0.787	1.000	0.787	drs	0.787	1.000	0.787	drs
H55	0.950	1.000	0.950	drs	0.921	0.971	0.948	drs	1.000	1.000	1.000	-	0.997	1.000	0.997	drs
H56	1.000	1.000	1.000	-	0.817	1.000	0.817	drs	0.893	1.000	0.893	drs	0.903	1.000	0.903	drs
H57	0.894	1.000	0.894	drs	0.856	0.909	0.942	drs	0.900	1.000	0.900	drs	0.912	1.000	0.912	drs
H58	0.871	0.996	0.875	drs	0.916	0.976	0.938	drs	0.961	1.000	0.961	drs	0.731	0.761	0.960	drs
H59	1.000	1.000	1.000	-	0.898	1.000	0.898	drs	1.000	1.000	1.000	-	1.000	1.000	1.000	-
H60	0.870	1.000	0.870	drs	0.887	1.000	0.887	drs	0.931	0.958	0.972	drs	0.940	1.000	0.940	drs
H61	1.000	1.000	1.000	-	0.801	0.802	0.999	irs	1.000	1.000	1.000	-	0.942	1.000	0.942	drs
Mean	0.939	0.997	0.942		0.898	0.952	0.943		0.934	0.992	0.941		0.922	0.977	0.945	

The symbol “-” denotes constant decreasing returns to scale, “drs” signifies decreasing returns to scale, and “irs” indicates increasing returns to scale.

### 3.2 Dynamic analysis of the Malmquist index for third-tier public hospitals in Guangxi

**Figure 3:**
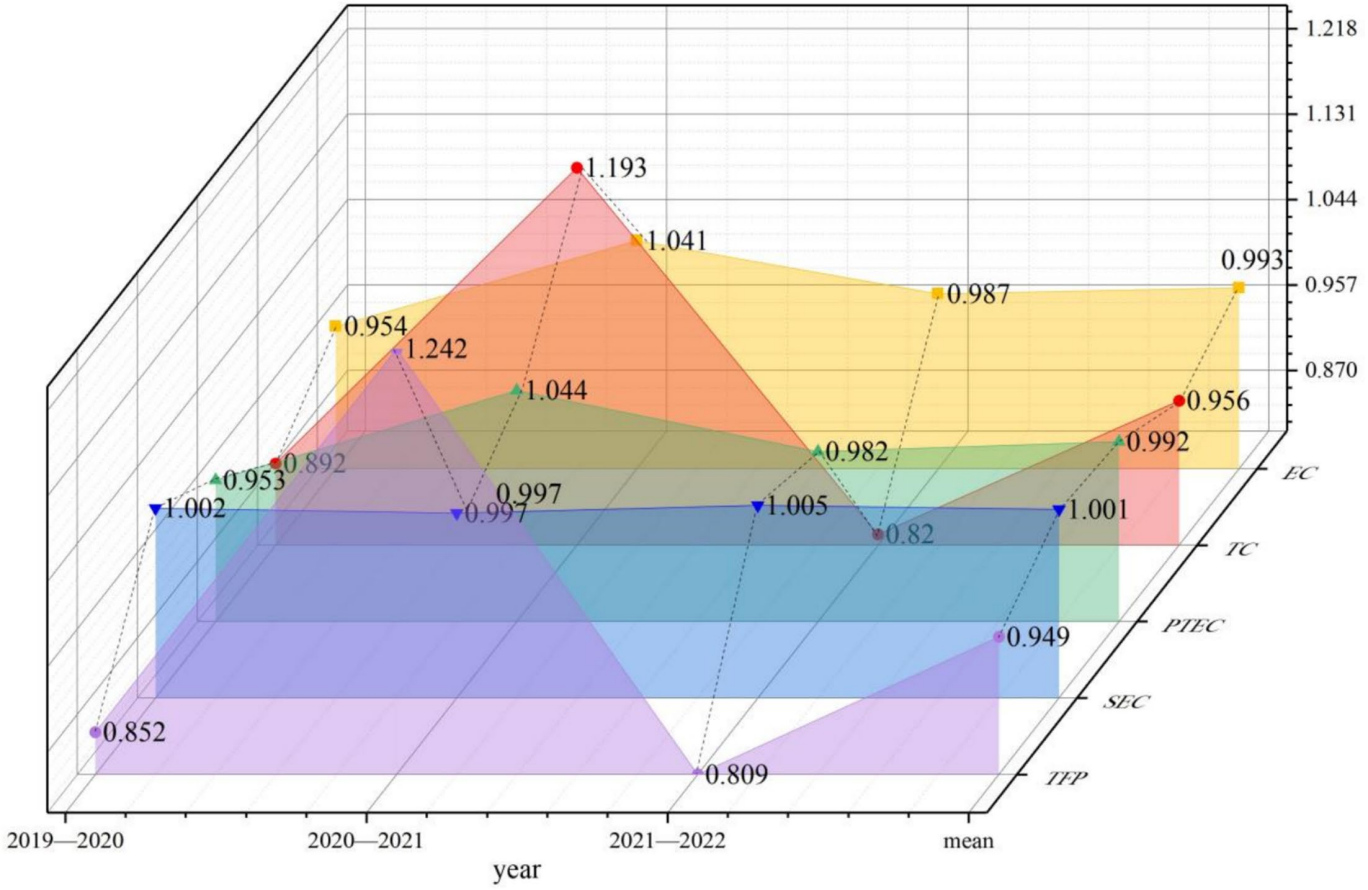
Dynamic efficiency value decomposition from 2019 to 2022 for Guangxi’s 61 tertiary public hospitals (EC stands for technical efficiency, TC for technological change, PTEC for pure technical efficiency, SE for scale efficiency, and TFP for total factor productivity).

**Figure 4:**
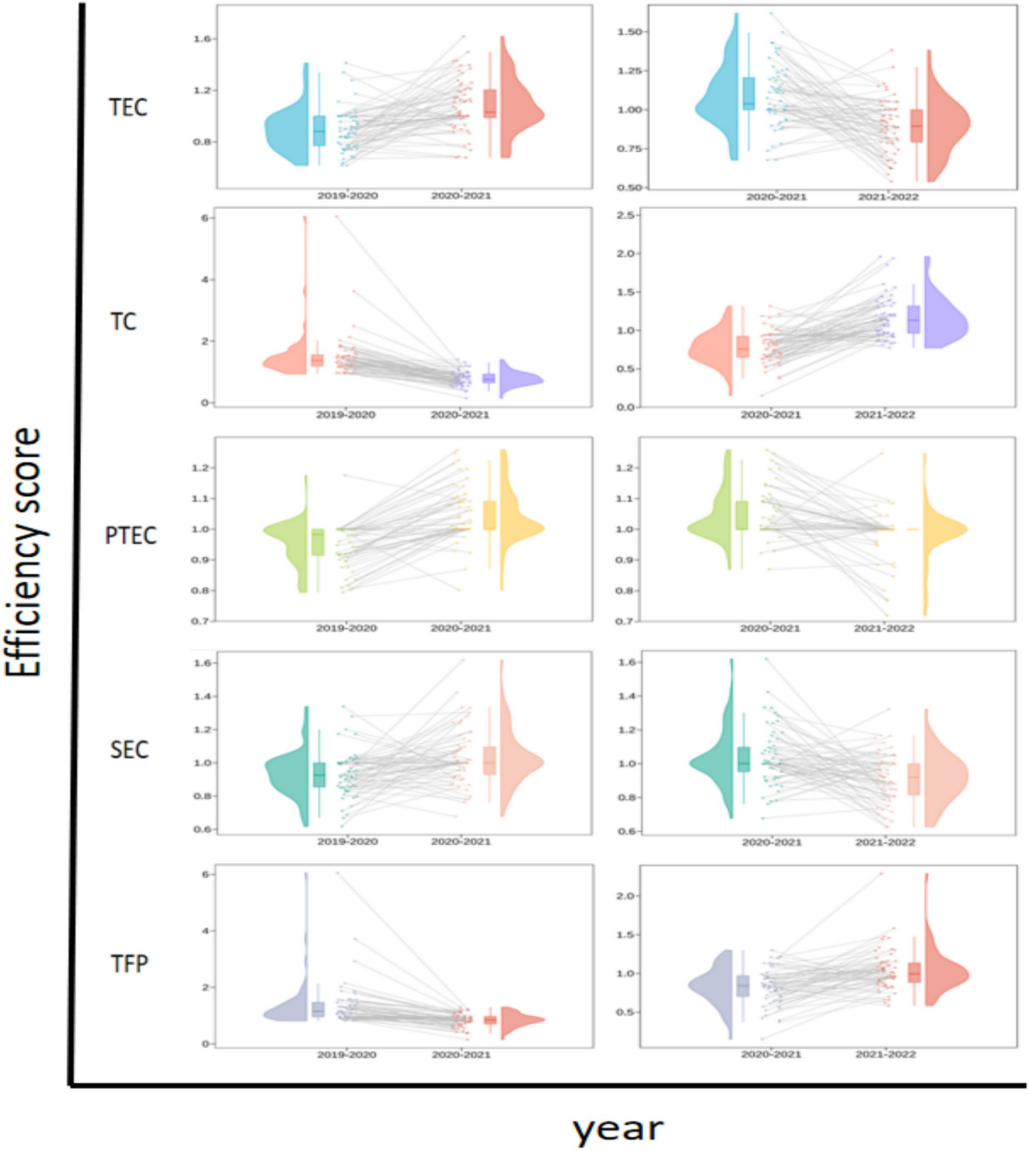
Changes in TFP and relative efficiency in secondary tertiary hospitals in Guangxi province from 2019 to 2022.

Overall, from a dynamic perspective, the Malmquist index decomposition indicators for sample hospitals from 2019 to 2022 demonstrated an initial increasing trend followed by a subsequent decline, as illustrated in Table 5 and Figure 3. The values for technical efficiency, TC, PTE and TFP that experienced a decrease from 2021–2022 compared to the period from 2020–2021 were recorded at 0.054, 0.373, 0.062 and 0.433, respectively. This suggests that resources are still significantly underutilized and a marked regression remains in comprehensive management and technological capabilities within the operation and management of tertiary public hospitals in Guangxi. Although SE was maintained at a level of 1, neglecting proper attention and effective control may result in subsequent issues concerning the rationality of the hospital production scale. From a microscopic perspective, 19 hospitals (31.15%) had TFP exceeding 1 over the observed years. Hospitals that demonstrated improvement solely attributable to the TC indicator included H6, H21, H32, H44, H50 and H59. Of the 42 hospitals exhibiting a TFP of less than 1, 13 (30.95%) experienced a decline in their TFP that was solely ascribed to a decrease in the TC indicator. Despite maintaining all other indicators at or above 1, six hospitals had a TFP of less than 1 owing to the influence of the TC indicator. The aforementioned situation reveals that the primary issue contributing to the decline in the operational management of third-tier public hospitals in Guangxi was significantly influenced by the TC indicator. Figure 4 displays this phenomenon in detail. Hospitals H9 and H35 had all indicators less than 1, which necessitated an increased focus on operational management.

**Table 5:** Malmquist index decomposition of 61 tertiary public hospitals in Guangxi from 2019 to 2022.

Hospital	TEC	TC	PTEC	SEC	TFP	Hospital	TEC	TC	PTEC	SEC	TFP
H1	1.000	0.526	1.000	1.000	0.526	H32	1.000	1.114	1.000	1.000	1.114
H2	1.026	0.921	1.000	1.026	0.945	H33	1.000	0.982	1.000	1.000	0.981
H3	1.000	0.926	1.000	1.000	0.926	H34	0.965	0.959	1.000	0.965	0.925
H4	0.949	1.000	1.000	0.949	0.950	H35	0.959	0.986	0.962	0.997	0.945
H5	1.000	0.877	1.000	1.000	0.877	H36	0.896	0.988	0.870	1.031	0.885
H6	1.000	1.101	1.000	1.000	1.101	H37	1.032	1.034	1.000	1.032	1.067
H7	1.000	0.630	1.000	1.000	0.630	H38	0.924	0.987	1.000	0.924	0.912
H8	1.000	0.813	1.000	1.000	0.813	H39	0.986	1.006	1.000	0.986	0.992
H9	0.941	0.869	0.953	0.987	0.818	H40	0.999	0.993	1.000	0.999	0.992
H10	0.934	0.938	1.000	0.934	0.876	H41	0.955	1.060	0.944	1.012	1.012
H11	1.057	0.941	1.000	1.057	0.995	H42	1.053	1.004	1.057	0.996	1.057
H12	1.000	0.868	1.000	1.000	0.868	H43	1.000	0.970	1.000	1.000	0.970
H13	1.024	1.034	1.000	1.024	1.058	H44	1.000	1.024	1.000	1.000	1.024
H14	1.018	0.957	1.000	1.018	0.974	H45	0.997	0.965	1.000	0.997	0.962
H15	1.000	0.937	1.000	1.000	0.937	H46	1.000	0.966	1.000	1.000	0.966
H16	0.944	0.980	1.000	0.944	0.925	H47	0.949	1.029	1.000	0.949	0.977
H17	1.027	0.982	1.000	1.027	1.008	H48	1.077	0.960	1.000	1.077	1.033
H18	0.913	0.928	1.000	0.913	0.847	H49	1.004	0.997	1.000	1.004	1.002
H19	0.999	0.947	1.000	0.999	0.947	H50	1.000	1.055	1.000	1.000	1.055
H20	0.962	0.935	1.000	0.962	0.900	H51	0.946	0.949	0.940	1.006	0.898
H21	1.000	1.009	1.000	1.000	1.009	H52	1.000	0.973	1.000	1.000	0.973
H22	1.000	0.905	1.000	1.000	0.905	H53	1.030	1.000	1.000	1.030	1.030
H23	1.018	0.951	1.000	1.018	0.968	H54	0.982	0.963	1.000	0.982	0.945
H24	1.003	0.952	0.965	1.039	0.954	H55	1.016	1.003	1.000	1.016	1.019
H25	1.011	0.940	1.000	1.011	0.951	H56	0.967	1.058	1.000	0.967	1.023
H26	1.040	0.930	1.000	1.040	0.967	H57	1.007	0.941	1.000	1.007	0.947
H27	1.000	0.958	1.000	1.000	0.958	H58	0.943	0.966	0.914	1.032	0.911
H28	1.041	0.965	1.000	1.041	1.005	H59	1.000	1.029	1.000	1.000	1.029
H29	0.980	1.001	0.941	1.041	0.981	H60	1.026	1.016	1.000	1.026	1.043
H30	1.031	0.933	1.000	1.031	0.962	H61	0.980	0.981	1.000	0.980	0.961
H31	1.023	0.995	1.000	1.023	1.018	Mean	0.993	0.956	0.992	1.001	0.949

### 3.3 Tobit regression analysis of tertiary public hospitals in Guangxi

In this study, the likelihood ratio (LR) test was conducted on the CE, PTE and SE values of 61 tertiary public hospitals in Guangxi. The *p*-values of CE, PTE and SE were 0.029, 0.000 and 0.023, respectively, all of which were below 0.05. This indicates that a mixed-effects Tobit model should be established. The results of the regression analysis on the influencing factors for CE indicated that variables such as bed size scale, the ratio of outpatient to inpatient patients and the percentage of discharged patients undergoing surgery within the dimension of functional positioning were statistically significant in the sample model. In specific terms, both the hospital scale and the ratio of outpatient to inpatient patients exerted a positive influence, whereas the percentage of discharged patients undergoing surgery had a negative effect. This suggests that tertiary hospitals should actively adjust their patient demographics and attach importance to treating patients requiring high-difficulty surgeries. From the perspective of operational efficiency, it was revealed in this study that business expenses exerted a negative influence on the CE of hospitals, whereas total annual revenue had a positive effect. These findings indicate that hospitals should prioritize controlling medical activity costs while increasing revenue and minimizing expenses. The results of the regression analysis on the factors influencing PTE indicated that the effects of all other indicators were not statistically significant except the bed size scale within the dimension of functional positioning. The results of the regression analysis on the factors influencing SE indicated that all the indicators within the dimension of functional positioning exhibited statistical significance for the sample model. Concretely, both the bed size scale and the ratio of outpatient to inpatient patients had a positive impact, while jurisdictional level and the percentage of discharged patients undergoing surgery among discharged patients exerted a negative influence. In the dimension of operational efficiency indicators, the business expenditure metric exhibited a negative impact, while the total annual revenue metric demonstrated a positive influence. The effects of sustainable development and satisfaction dimension indicators on SE showed no statistical significance. Please refer to Table 3 for further details.

## 4 Discussion

### 4.1 The SE indicator reveals inappropriate hospital production scale

The results of the BCC static model demonstrated that the fluctuation and decline in CE among sample hospitals were primarily driven by SE from 2019 to 2022. From the micro perspective of various DMUs, SE emerged as the main factor influencing overall efficiency. Among hospitals with a CE value of less than 1 during this period, those rendered DEA-ineffective because of invalid SE were numbered 27, 12, 30 and 29, respectively, for each year, which accounted for 87.10, 28.57, 76.92 and 78.38%, respectively. Except for the year 2020, these proportions consistently exceeded 75%. Furthermore, more than half of DEA-ineffective hospitals observed over these years operated under DRS, which indicated a certain risk of excessive expansion among tertiary public hospitals in Guangxi. This result was consistent with that reported by Kirigia and Asbu, who suggested that most hospitals suffer from low efficiency on account of inappropriate scale (44). A detailed analysis of specific survey data revealed that some hospitals saw a continuous increase in inputs like the number of healthcare personnel and actual open bed counts in several years. However, key output indicators including the proportion of medical service revenue, debt-to-asset ratio, medical surplus rate and the proportion of personnel expenditures did not improve alongside hospital size expansion or increased resource input. With the expansion of the hospital scale, operational costs also increased.

Therefore, it is critical to fully leverage the “conductor’s baton” role of performance assessment policies in tertiary public hospitals. This can promote a shift in the development model of public hospitals from scale expansion to quality and efficiency and in their management model from extensive administrative management to comprehensive performance management.

### 4.2 The TC indicator is key to improving the operational efficiency of hospitals

The Malmquist dynamic analysis results indicated that the TFP of 61 public tertiary hospitals in Guangxi was primarily influenced by a combination of TC, TEC and SEC. From a positive perspective, it can be observed that 31.59% of the hospitals experienced an increase in TFP solely due to improvements in the TC indicator. Conversely, from a negative standpoint, 30.95% of the hospitals saw a decline in TFP exclusively owing to changes in the TC indicator. It is worth noting that none of the TEC, PTEC and SEC independently contributed to a decline in the TFP of these hospitals. Despite the progress made by hospitals H2, H11, H14, H23, H25 and H30 in other indicators, the decline in the TC index led to a decrease in TFP. This indicates that the TC index is crucial for improving the operational efficiency of tertiary public hospitals in Guangxi. It suggests that some hospitals are encountering challenges like outdated operational management techniques and methods, and the inefficient use of funds. This result was in line with that reported by Chen (45), who believed that the TC indicator should be the priority direction for improvement to improve the operational efficiency of hospitals. Through field investigations, it has been found that tertiary public general hospitals in Guangxi mostly have no separate operational departments. Instead, the financial department is primarily responsible for managing hospital operations. This arrangement makes it challenging to assign specific responsibilities to corresponding departments or individuals during actual work processes, which results in low operational efficiency and difficulties in implementing refined management practices. In addition, some hospitals are confronted with issues where existing information systems fail to satisfy operational management needs. For the time being, a majority of hospital operation management information system modules concentrate mainly on basic functions such as accounting, cost management and asset management. Only a few hospitals possess modules for risk control, operational analysis and project management related to scientific research and education. More than that, compatibility issues also exist between different systems within hospitals that pose technical barriers to the implementation of refined management practices.

In light of the aforementioned issues, on the one hand, healthcare institutions ought to establish a structured operational management system and standardize the creation of operational management departments to enhance operational efficiency. On the other hand, they must advance the informatization of operational management and facilitate a transition from the traditional “process-driven” model to a “dual-driven” approach integrating both data and processes.

### 4.3 The imbalance in the operational development of hospitals remains a concern

According to the analysis of the Malmquist dynamic model, six (31.58%) provincial-level hospitals had a TFP of greater than 1 from 2019 to 2022. At the municipal level, 11 hospitals (57.89%) achieved a TFP of greater than 1. At the county level, only two hospitals (10.53%) obtained a TFP of greater than 1. This reflects the unbalanced operational development among hospitals of different jurisdictional levels. From a micro perspective, the M index indicated that five tertiary public hospitals in Nanning four in Guilin, three in Liuzhou and two each in Hechi and Yulin across various cities had a technical efficiency value of greater than 1. Beyond that, Wuzhou, Beihai, Laibin and Guigang each have one such hospital. The hospitals in the remaining cities experienced no growth in their TEC indicators. This further demonstrates the imbalance in the operational development of tertiary public hospitals across different regions of Guangxi. The exploration of underlying factors showed that Guangxi exhibits a relatively weak economic foundation featuring disparities in economic development across multiple regions and an imbalance in health financial investment. These conditions inevitably lead to differences in the operational development of hospitals. During the observation period, Guangxi grappled with the COVID-19 pandemic, which had varying effects across different areas. The responsibilities associated with epidemic prevention and control, nucleic acid testing tasks, the consumption of protective materials and labor expenses for dispatched medical staff differ among hospitals of varying levels as well. These external factors further influence hospital operations to differing degrees. A study by Androutsou and Lupu also demonstrated that COVID-19 exerted a huge impact on the operational efficiency of hospitals (46, 47).

As a result, hospitals should start from the actual situation of resource allocation to enhance resource utilization rates. Concurrently, they must strengthen cost management and accurately control the “revenue generation” and “cost saving” of hospitals to maximize operational management benefits. In addition, it is suggested that hospital management departments consciously balance the disparities in inputs among hospitals within a region, formulate rational regional health plans, and supervise and regulate hospital operational efficiency (48).

### 4.4 Structural adjustment supports the advancement of hospital operations to a higher level

The Tobit regression analysis results demonstrated that the proportion of surgical patients discharged from hospitals had a negative impact on the operational efficiency of 61 third-level public hospitals in Guangxi. This finding contradicts conventional empirical judgments. Nevertheless, it is important to note that all the sample hospitals included in this study are classified as third-level or above public hospitals in China, and specifically defined as institutions primarily focused on treating complex and challenging diseases and emergencies. Therefore, they need to receive patients with higher treatment difficulty (49). An investigation into the quadruple-level surgery situation of sample hospitals revealed that Hospital H7 consistently had the highest proportion of level 4 surgeries from 2019 to 2022, with ratios of 42.79, 41.71, 37.11 and 41.74%, respectively. The hospital with the lowest proportion was H42 in 2019, with a ratio of 0.05%, while Hospital H58 had the lowest proportion from 2020 to 2022, with ratios of 0.14, 0.42 and 0.37%. In 2019, 2020 and 2022, only Hospital H7 in Guangxi reached the full score value (≥ 40%) for level 4 surgeries as per the “National Performance Assessment Full Mark Value” for tertiary public hospitals. In 2021, however, no hospitals in Guangxi achieved this full score value. This indicates that the patient intake structure of hospitals and their capacity to provide high-difficulty medical services still have room for adjustment. The business expenditure indicator also had a reverse effect on the operational efficiency of sample hospitals. The business activity expenses of hospitals encompass labor wages, fixed asset depreciation, costs of drugs and consumables, official expenses, etc. When controlling business expenditures, hospitals should also abide by national guidelines and reasonably increase personnel expenditures to retain talents. Research data indicate that the hospital with the highest proportion of personnel expenditures was consistently H6 over the four years, with values of 70.52, 72.05, 74.76 and 74.12%. In 2019, the hospital with the lowest proportion of personnel expenditures was H36, with a ratio of 28.3%. In 2020, the hospital with the lowest proportion of personnel expenditures was H53, with a ratio of 29.51%. Between 2021 and 2022, the hospital with the lowest proportion of personnel expenditures was H35, with ratios of 28.65 and 31.44%, respectively. The number of hospitals failing to reach the “national performance assessment full mark value (38.09%)” for tertiary public hospitals in the four years were 34 (55.74%), 31 (51.67%), 28 (46.67%) and 18 (30.00%), respectively. While the personnel expenditures of various hospitals continued to increase, a significant portion of hospitals remained unable to achieve the full mark value set by the national assessment.

Therefore, hospitals should actively align with the tiered diagnosis and treatment system by adjusting the admission structure for surgical patients and gradually referring common and frequently occurring diseases, as well as patients in the stable or recovery phase, to lower-level medical institutions. They should also progressively lower the proportion of outpatient visits for common ailments at urban tertiary general hospitals. This is aligned with the performance assessment goals for national tertiary public hospitals (27). Meanwhile, hospitals should vigorously develop new technologies and projects, keep enhancing their capacity to provide high-complexity medical services, strengthen their radiating capabilities and improve the quality and level of social services.

## 5 Limitations

This study has several limitations: First, it only secured data from 2019 to 2022, which is a relatively short period. Nonetheless, the findings remain beneficial to assessing the short-term impact of hospital inefficiency. It is necessary to monitor data over a greater number of years for further validation in the future. Second, the evaluation was confined to tertiary public hospitals, and the authors had no access to data from secondary or primary hospitals. Thus, it is essential to evaluate the operational efficiency of hospitals at other levels in future research. Third, only hospitals in Guangxi were evaluated, which limited the generalizability of the findings. This limitation is common in research, but hospitals from this province were fortunately included in this study. Moreover, the province studied represents the western regions of China in terms of average economic and social development levels. Therefore, the findings remain applicable to public hospitals in the western regions of China.

## 6 Conclusion

In the present study, the operational efficiency of 61 hospitals in Guangxi was analyzed, and the factors influencing the operational efficiency of hospitals were explored from macro and micro perspectives. The results indicated that tertiary public hospitals in Guangxi have relatively low overall operational efficiency, with most facing the issue of excessive scale expansion. Tobit regression analysis showed that bed size scale, the proportion of discharged patients undergoing surgery, business expenditures and total annual revenue affected hospital operational efficiency to a large extent. Public hospitals should consider the economic conditions of the region while seeking development, and ensure both efficient operation and sustainable development.

## Data Availability

The original contributions presented in the study are included in the article/Supplementary material, further inquiries can be directed to the corresponding authors.
